# Breaking the silence: The role of extracellular vesicles in unraveling the diagnosis and treatment of endometriosis

**DOI:** 10.20517/evcna.2023.43

**Published:** 2023-12-04

**Authors:** Kumar Utkarsh, Namita Srivastava, Christopher Papayannakos, Ashima Nayyar, Azhar Khan, Shabirul Haque

**Affiliations:** ^1^Department of Microbiology and Biotechnology, Shoolini University, Solan, Himachal Pradesh 173229, India.; ^2^Department of Pediatrics, Institute of Molecular Medicine, Feinstein Institute for Medical Research, Northwell Health, 350 Community Drive, Manhasset, New York, NY 11030, USA.; ^3^Department of Neurology, Robert Wood Johnson Medical School, Rutgers University, New Brunswick, NJ 08901, USA.; ^4^Department of Autoimmune Diseases, Institute of Molecular Medicine, Feinstein Institute for Medical Research, Northwell Health, 350 Community Drive, Manhasset, New York, NY 11030, USA.

**Keywords:** Endometriosis, extracellular vesicles, apoptotic bodies, microvesicles, exosomes

## Abstract

Cell-to-cell communication is believed to be facilitated by membrane-bound vesicles called extracellular vesicles (EVs), which are released by cells. Protein, lipids, and nucleic acids are major cargo of EVs and are transported in these vesicles. Depending on the parent and recipient cell types, they can affect a wide variety of biological processes in the tissues to which they are delivered. EVs are essential for embryo implantation and endometriosis, and they are located in the uterine cavities of different species, where they promote blastocyst and endometrial preparation for implantation. This review focuses on what is currently understood regarding pathologic and diagnostic characteristics, and the potential therapeutic value of EVs in the context of endometriosis, where they can be used for drug delivery and targeted therapy due to their ability to carry bioactive molecules to specific cells or tissues. The findings of this review highlight the potential for a wide range of clinical applications that involve endometrial EVs in the areas of treatment, such as surgical and pharmacological, diagnostic biomarker development, and drug delivery systems, all with the ultimate goal of improving pregnancy success rates.

## INTRODUCTION

Endometriosis is an inflammatory condition caused by aberrant growth of uterine tissue outside the uterus, which is driven by its own peritoneal environment^[1]^. Despite decades of study, millions of women around the world still suffer from chronic pelvic pain and infertility caused by endometriosis^[2]^. Multiple studies have shown that women with endometriosis are more likely to experience retrograde menstruation, which occurs when period blood flows back into the fallopian tubes and pelvic cavity instead of exiting the body through the vagina^[3]^. Endometrial fragments were first proposed as the cause of endometriosis in Sampson's theory of retrograde menstruation, which inspired further investigations^[4]^.

There are five subtypes of endometriosis, including: endometriosis of the ovary, endometriosis of the deep peritoneum, endometriosis of the superficial peritoneum, retro-cervical endometriosis, and bladder endometriosis^[5]^. While estimates of endometriosis prevalence in the general population range widely, one meta-analysis from 2020 suggests rates of 3%-10% and annual incidence rates of 2-7/1000 women^[6]^. Women between the ages of 25 and 35 are most likely to be diagnosed with endometriosis, which is uncommon in premenarchal and menopausal women^[7]^. Common initial symptoms of endometriosis are dysmenorrhea, dyspareunia, pelvic pain, and infertility^[8]^. In addition to abnormal uterine bleeding, other clinical manifestations include noncyclic lower abdominal pain, dysuria, bladder or ureteral complications, and gastrointestinal symptoms such as dysphasia, tenesmus, and intestinal perforation^[9]^. Endometriosis has also been linked to ovarian cancer and obstetrical complications such as infertility, ectopic pregnancy, and preterm birth. Women with endometriosis may have a reduced chance of conceiving due to the formation of scar tissue and adhesions in the pelvic region that can interfere with the fertilization of an egg, which has been widely reported in the literature^[10]^. Clearly, there is a great need for a deeper understanding of the development and pathology of endometriosis, and EVs represent a dynamic biology that offers new opportunities for diagnostics and therapeutic intervention.

## ENDOMETRIOSIS: AN OVERVIEW

Endometriosis is a progressive disease with pathogenic mechanisms that are like "low-grade cancer" in that they can invade tissue and become metastatic. Endometriosis lesions can invade and infiltrate surrounding tissues, similar to the way cancer cells spread, and may also produce factors that promote angiogenesis, or the formation of new blood vessels, to support their growth and spread^[11]^. If untreated or undiagnosed, these conditions may deteriorate over time and give rise to complex disease patterns that are distinct^[10]^. During the menstrual cycle, hormonal fluctuations cause lesions to grow, shed, and bleed^[12]^. The result is believed to be inflammation and irritation of surrounding tissues, which leads to adhesions. Endometriosis is a benign gynecological condition that is persistent, painful, and hormone-dependent^[8]^. Although the root cause of endometriosis is unknown, Sampson's theory of retrograde menstruation offers a possible explanation for the development of the condition. In this theory, it has been proposed that menstrual discharge is reabsorbed into the peritoneal cavity during uterine contraction, where it adheres to tissue and can cause ectopic lesions^[13]^. Only 5% to 10% of patients with retrograde menstruation are later diagnosed with endometriosis^[14]^. Studies have shown that endometriosis could be potentially caused by a defective immune response that encourages inflammatory reactions and angiogenesis^[15]^.

The disease can manifest in several ways. Superficial Endometriosis is the most common type of endometriosis and involves the presence of small patches or lesions of endometrial tissue on the surface of pelvic organs such as the ovaries, fallopian tubes, and the peritoneum (the lining of the abdominal cavity). These lesions are often referred to as “implants”. This type of endometriosis is also known as peritoneal endometriosis^[16]^. Deep Infiltrating Endometriosis (DIE) infiltrates deeper into the pelvic organs and structures, including the bowel, bladder, and uterosacral ligaments. These lesions can be more extensive and cause severe pain and complications, such as bowel or bladder obstruction^[17]^. Adenomyosis, while not always considered a type of endometriosis, involves the growth of endometrial tissue into the muscular wall of the uterus. This condition can lead to an enlarged, tender uterus and is often associated with heavy menstrual bleeding and pain^[18]^. Ovarian Endometriomas (Endometriotic Cysts) are cysts that form on the ovaries and are filled with endometrial tissue and blood. Ovarian endometriomas can vary in size and cause pelvic pain, ovarian cyst rupture, and fertility issues^[19]^. Retro-cervical Endometriosis involves the presence of endometrial tissue behind the cervix (the lower part of the uterus). It can cause pain during intercourse, pelvic pain, and discomfort during bowel movements^[20]^. Finally, Bladder Endometriosis affects the bladder, causing symptoms such as urinary urgency, frequency, and discomfort during urination^[21]^.

Histopathological analysis is used to identify cases of endometriosis^[22]^. However, besides a comprehensive medical history and gynecological examination with a speculum, imaging techniques, laparoscopy, and biochemical tests are helpful^[23]^.

Ultrasound examinations serve as the foundation of endometriosis diagnosis^[24]^. Ultrasound examination (USG) is a helpful diagnostic tool to diagnose ovarian endometrial cysts and other congenital defects that promote the retrograde flow of menstrual blood into the abdominal cavity^[25]^. Endometriosis infiltrating the urinary bladder or large intestine justifies cystoscopy, colonoscopy, and transrectal ultrasound^[25]^. Transvaginal Sonography (TVS) is also useful in cases of endometriosis with extensive infiltration^[26]^. Using water contrast, we can locate intestinal foci and gauge their development. In patients with deep infiltrating endometriosis, endometrial cells are found more than 5 mm below the peritoneal surface^[27]^. Adhesions and distortions in pelvic anatomy are brought on by severe lesions, and they most commonly occur in the posterior pelvic compartment, which includes the rectovaginal septum, posterior vaginal vault, uterosacral ligaments, and the anterior rectal wall^[28,29]^. Dysmenorrhea, dyspareunia, chronic pelvic pain, dystrophy, and dyschezia are symptoms of dysfunction of the pelvic organs and the pelvic floor muscles (PFM), which can cause major changes in addition to the basic DIE pain syndrome^[30]^.

Women who suffer from chronic pelvic pain are more likely to develop nonrelaxing PFM due to factors such as pelvic muscle injuries (both direct and indirect (neuropathic)), pelvic pain (both acute and chronic), and inflammation. When evaluating pelvic floor muscles, palpation and electromyography both cause discomfort and spasms. Using transperineal ultrasound to evaluate pelvic floor morphometry is a safe, reliable, and noninvasive option^[31,32]^. The ultrasound examination is the primary diagnostic tool for this disease, although magnetic resonance imaging (MRI) is also helpful. Histopathological confirmation during laparoscopic surgery for endometriosis is considered the gold standard for diagnosis^[33]^. Because endometriosis is so rarely found in the vaginal vault and cervix, this area must be thoroughly examined as part of any gynecological examination. Uterine retroflection, which is commonly induced by intraperitoneal adhesions, is a frequent and defining feature of endometriosis patients^[34,35]^.

## CLINICAL MANAGEMENT AND TREATMENT

The most effective treatment for endometriosis would target both the hormonal and immunologic settings simultaneously. It should suppress endometriotic cell proliferation and progression while increasing apoptosis. Treatment for endometriosis should be symptomatic, targeted to endometriotic tissue only, normalize the invasive mechanisms, and control the growth of new blood vessels in the lining of the uterus (endometrial neuro-angiogenesis). There is no "gold standard" treatment and most doctors mistakenly believe endometriosis to be a local condition when it is, in fact, more likely to be a systemic condition and most treatment options vary depending on the severity of the condition, the age and health of the patient, and other factors^[36]^. Various diagnostic plans such as expectant management, medical therapy, conservative surgical intervention, and combination therapy, which can include pre- and post-operative medical treatment, are just some of the various courses of action described^[37]^. Several non-curative medical treatments such as hormonal therapy, pain management, surgery, and complementary and alternative therapy for endometriosis have been developed, which are aimed at slowing disease progress^[38]^. During conservative surgery, the patient's pelvic and abdominal structures are repaired with active endometrial lesions removed, and any fibrous adhesions are cut.

Endometriosis is a chronic condition with serious medical implications and a difficult treatment process. Effective treatment for endometriosis can be achieved through pharmaceutical, surgical, or a combination of these methods. Women who notice health problems in themselves report them to their doctors, allowing for the planning of appropriate treatments. The presence of pain does not prevent patients from waiting too long to seek medical attention, which can lead to complications and the need for extensive surgery. Endometriosis is treated with pharmaceutical, surgical, and combination therapies^[39]^. Currently, hormonal therapy is permitted for three months even if there is no histopathological proof of the illness^[40]^. Factors such as age, fertility goals, severity of symptoms, and diagnosis of endometriosis all play a role in determining the best course of treatment. A healthy diet and way of life are also important in this disease.

Pharmacological treatment is a type of drug therapy that reduces pain, stops endometrial foci growth, restores fertility, relieves endometriosis symptoms, and enhances reproductive outcomes by targeting pain, inflammation, endometrial development, and fertility. There are several classes of drugs and their modes of action mentioned here. We discuss each drug in brief. NSAIDs decrease prostaglandin synthesis to lessen inflammation and discomfort^[41]^. Danazol, a testosterone derivative, inhibits GnRH, reducing pituitary gland LH and FSH output. The most common side effects are fluid retention, weight gain, breast sensitivity, spotting, irregular genital bleeding, and vaginal dryness. Estrogen-progestogen oral contraceptives reduce FSH and stabilize the endometrium, minimizing discomfort. Intrauterine devices release levonorgestrel. Aromatase inhibitors are the latest endometriosis treatment; they take advantage of endometriosis cells' unique activity^[42]^. Aromatase inhibitors reduce estrogen by inhibiting endometriosis tissue and follicle estrogen production. The cruelest side effect is osteoporosis, which causes bone calcium loss. Other side effects symptoms include insomnia, libido loss, and vaginal dryness^[41]^. Elagolix was approved by the FDA to treat moderate to severe endometriosis pain^[43]^. This medicine reduced pelvic and sex-related endometrial pain (150 mg once/day or 200 mg twice/day)^[44]^. The higher dose (200 mg/day) decreases bone density. This drug must be taken via oral route^[45]^. Elagolix is a GnRH antagonist without peptides, which decreases estrogen levels in a dose-dependent manner and provides long-lasting pain relief for moderate to severe endometriosis^[46]^. The use of elagolix is associated with a modest but actual risk of bone loss, but its severity does not affect future fracture risk, as evidenced by literature^[47,48]^. Overall, a summary of endometriosis medical treatment is tabulated [Table 1].

**Table 1:** Medical treatment of endometriosis

**SL.no**	**Class**	**Drug**	**Pain-relieving action**
	Progestins	Etonogestrel-releasing implant	Atrophy or decidualization of lesions
		Norethindrone acetate^+^	Angiogenesis inhibition
		Levonorgestrel releasing IUS	The inhibition of matrix metalloproteinase aided in the development and implantation of ectopic endometrium.
		Medroxyprogesterone acetate	
		Dienogest*	
	Androgenic steroids	Danazol^+^	Pituitary gonadotropin secretion suppression
			Inhibits regional expansion
			estrogenic enzyme suppression
	Estrogen–progestin concentration	Monophasic estrogen-progestin^+^	lesions' dedifferentiation or atrophy
	GnRH agonists	Leuprolide depot^+^	Reduced ovarian steroidogenesis as a result of gonadotropin secretion inhibition
		Goserelin^+^	
		Nafarelin^+^	
	GnRH antagonists	Elagolix	Reduced ovarian steroidogenesis as a result of gonadotropin secretion inhibition
	Antiandrogens	Cyproterone acetate^*^	Competition-based androgen receptor inhibition
	Aromatase inhibitors	Letrozole	local inhibition of aromatase-mediated testosterone to estrogen conversion
		Anastrozole	
	Specific progesterone receptor activators	Mifepristone	Progesterone receptor agonist or antagonist-mediated ovulation inhibition
		Ulipristal acetate	

+: U.S. Food and Drug Administration-approved for endometriosis; *: Used as monotherapy.

**Surgical Treatment**: It is possible to use minimally invasive or radically invasive surgical techniques. The treatment of adolescents and women who would want to become pregnant would have fewer options available to them as clinicians would try to preserve as much of the reproductive system as possible so that they could go on to have children. Patients who do not want to have children or who have persistent pain despite medication are often subjected to invasive surgical procedures. The following are some of the situations in which endometriosis surgery is required: pelvic pain, infertility due to endometriosis, and ovarian endometrial cysts. Regardless of the severity of endometriosis, laparoscopy is the preferred surgical method for the removal of the disease^[49]^. The combination of surgical and pharmacological treatment yields the most effective therapeutic results. Medications are administered both before and after surgical procedures. Endometriosis with deep infiltration is acceptable to resection via laparoscopy with the assistance of a robot, particularly in the rectal-sigmoid region. Endometriosis is best treated with a minimally invasive laparoscopic or laparoscopic robot-assisted procedure^[50]^. However, complications involving the gastrointestinal, urinary, or sexual tract functions frequently arise when DIE (Deep Infiltrating Endometriosis) lesions are surgically removed^[51]^. Rectal fistula (0.3%-2%), intestinal stenosis (2%), and bladder atony (4%-6%) are all surgical complications that can arise after DIE^[52-54]^. Endometriosis has been the subject of several comparative studies^[55-57]^ between laparoscopic and robotically assisted surgical procedures. Pain relief after laparoscopically assisted robotic surgery tends to last longer than pain relief after laparoscopic surgery^[58,59]^, though these findings are questionable. While both robot-assisted and traditional laparoscopies have their advantages, previous studies have shown conflicting results. However, robotic surgery may be a better option for patients with complex pelvic conditions like severe endometriosis, a high body mass index, or multiple previous surgeries^[60]^. In this study, researchers found no statistically significant differences between robotic and laparoscopic surgery in terms of perioperative outcomes. Some of these studies have demonstrated that robotic-assisted procedures require longer surgical times than laparoscopy, while others have demonstrated the benefits of robotic surgery^[61]^. In cases of isolated sting-rectal DIE, robot-assisted nerve-sparing laparoscopic rectal nodulectomy has been shown to be an effective and safe treatment option^[62]^. The most recent research suggests that DIE resection via robot-assisted laparoscopy is attainable^[63]^.

Over the years, several surgical methods have been described, including laser ablation, Electrocautery, radiofrequency ablation, and Excision with bowel or bladder resection, but none of them have proven to be noticeably superior. It is important to note that research into endometriosis treatment is ongoing and that the wide range of medical treatments including nonsteroidal anti-inflammatory drugs (NSAIDs), hormonal therapies such as birth control pills and gonadotropin-releasing hormone (GnRH) agonists, and newer targeted therapies such as aromatase inhibitors and selective progesterone receptor modulators (SPRMs) are available^[38]^. However, they show that no single approach is ideal for every patient. Each patient requires a unique approach to therapy. Most current medical treatments for endometriosis aim to induce a hypoestrogenic state by means of chronic anovulation, a postmenopausal hormonal profile, or pseudopregnancy-inducing decidualization of endometrial tissue, even though the pathogenesis of the disease is poorly understood. Medical treatments for endometriosis include NSAIDs (nonsteroidal anti-inflammatory drugs), hormonal therapy, combined hormonal contraceptives (CHO), progestins (oral, parenteral, or intrauterine route), gonadotropin-releasing hormone agonists (GnRHa), danazol, and aromatase inhibitors^[64]^.

## CHARACTERIZATION AND BIOSYNTHESIS OF EXTRACELLULAR VESICLES

EVs are membrane-bound vesicles that are released by cells and can be found in bodily fluids such as exosomes, microvesicles, and apoptotic bodies^[65]^. While EVs are classified mainly on their site of biogenesis, they possess partially overlapping physical and biochemical characteristics. These membrane vesicles are released into the extracellular environment in response to specific stimuli under both normal and pathological conditions^[66,67]^. Researchers struggle to collect and study consistent EV samples because of a lack of diagnostic markers and isolation procedures^[68]^. It is possible to separate EVs using a variety of methods. Methods such as ultrafiltration, differential centrifugation, density gradient centrifugation, chromatography, immunological capture, and precipitation are just a few examples. The efficiency with which soluble components can be isolated from EVs and the size of EVs that can be isolated differ across methods^[69]^. Consequently, we follow the convention to utilize the broader term of EV when discussing EV biology.

Exosomes are small spherical EVs delineated by a lipid bilayer, ranging from 30-150 nm in diameter. When the limiting membrane of a late endosome buds inward, it forms a Multivesicular body (MVB) that contains intraluminal vesicles (ILVs), and when an MVB fuses with the cell’s plasma membrane, it releases the ILVs as exosomes^[70]^. The MVBs can undergo a variety of cellular fates, one of which is the fusion of the limiting membrane with the plasma membrane, which results in the release of its ILVs into the extracellular spaces^[71]^. Once there, they are termed exosomes and are free to distribute throughout extracellular space and interact with recipient cells^[69]^. An initial description of this mechanism was described during reticulocyte maturation *in vitro*^[72]^. Microvesicles are larger EVs with a diameter spanning from 100–1,000 nm. Microvesicles, unlike exosomes, form when the plasma membrane bulges outward in response to passive cellular events^[73]^. Apoptotic bodies are heterogeneous vesicles containing organelles and nuclear fragments and have sizes ranging from 1.0 to 5.0 nm^[74]^. Currently, novel EV subpopulations are being described. Referred to as exomeres, they are exosome-like vesicles with diameters of 60-80 nm and 90-120 nm^[75]^. According to the study, each subset of nanoparticles has a unique biological purpose because of their varied biodistribution patterns^[75]^. Therefore, understanding the unique biodistribution patterns and biological functions of different subsets of nanoparticles is essential for developing effective nanotechnology-based therapies for endometriosis.

**Figure 1:**
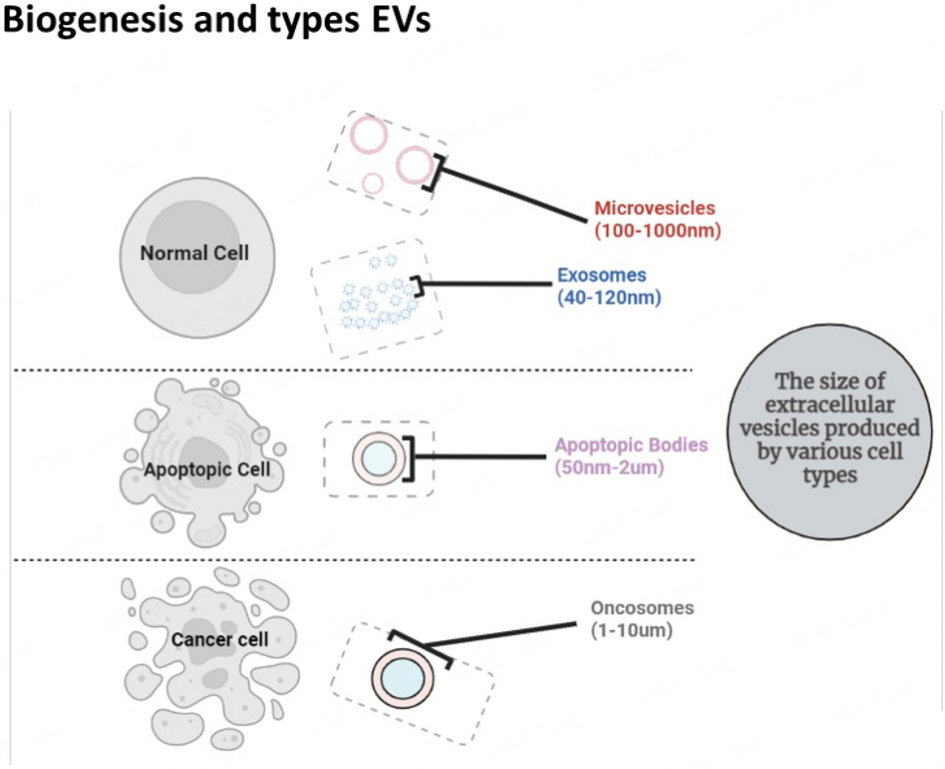
Schematic diagram distinguishes between microvesicles, apoptotic bodies, and oncosomes. All three are major types of extracellular vesicles. It illustrates the sizes of different extracellular vesicles that are produced by various cell types.

As demonstrated schematically [Figure 1], exosomes and microvesicles have been extensively studied and have been shown to play significant roles in biological processes. Biologically active substances such as proteins^[76]^ and lipids^[77]^ are selectively packaged to the sites of EV biogenesis. Parental cell releasing the EVs communicates with the cells via (1) Activation of target-cell intracellular signaling through interaction between membrane proteins; (2) exosomal membrane protein cleavage near receptors on target cells; (3) content transfer from EVs to target cells via fusion; and (4) Recipient cell phagocytosis of EVs^[78]^. The EVs can be extracted from extracellular fluid from the culture media or the body fluids^[79]^. There is always debate over various isolation techniques. The optimal method relies on the nature of the research question, the nature of the application, the purity required, and the desired concentration^[80]^. The proper methods for EV isolation and analysis have been established by the International Society for Extracellular Vesicles (ISEV)^[80]^. There are several methods, such as ultracentrifugation, filtering, polymer precipitation, immunoaffinity, and microfluidic methods, are frequently used to isolate EVs, all leading to varying degrees of yield and purity^[81]^. The characterization EV can be carried out using several methods such as flow cytometry, electron, and atomic force microscopy (gold standard for morphological characterization), optical particle tracking, and western blotting to assess the protein content, morphology, size, concentration, and purity, respectively^[82]^.

## CHALLENGES IN CURRENT EXTRACELLULAR VESICLE STUDIES

Despite increasing scholarly interest in the field, research on EVs and their effects on reproductive health in women varies in terms of both quantity and quality^[83]^. Various EV studies have examined EVs of varying sizes and with varying sets of components due to the use of varying methodologies^[84,85]^. Any future studies involving EVs should adhere to the updated 2018 EV minimal information criteria (MISEV2018). For more accurate and thorough coverage of EV science, these standards include both linguistic and technical suggestions^[86]^. The concentration, content, and function of EVs, as well as their subsequent analysis, may be affected by the collection, isolation, and storage of biofluids^[87]^. The viscosity and protein content of these fluids vary, necessitating specific methods based on the biofluid or tissue studied^[88]^. While a range of strategies for balancing recovery and EV specificity are now available, research to date has been hampered by a lack of technique standardization, which is critical for minimizing artifacts^[85]^. Additionally, to reduce impurity, embryos for EV research should be incubated in a serum-free medium^[89]^. If this is not possible, then EVs should be isolated from serum and used as a control in culture media.

The storage of the vesicles is an important factor that influences the findings of EV research. It is advised against freeze-thaw as it breaks vesicles, exchanging their soluble content with extracellular milieu pellets at -80 °C, which can lead to crystal formation, cryo-precipitation, and vesicle destruction^[90]^. In addition, the aliquots should not go through multiple freeze-thaw cycles before being analyzed^[91,92]^. Using protective agents, such as ion additives, to mitigate the osmotic pressure is crucial during EV isolation for the cargo being examined^[93,94]^. The compiled information gives an overview of challenges in reproductive EV research[Table 2].

**Table 2:** Challenges in Extracellular vesicles research

**SL. No**	**EV-related procedures**	**Challenges**
	EV collection	The type of (body) fluid, the nature of EVs of interest and their biological origin, as well as the downstream analysis, all influence the need for an optimal methodology
	Various EV isolation techniques	To avoid variations in the concentration and size of EVs obtained and evaluated, there is a requirement for uniformity in the techniques for EV separation
	Storing EV	To prevent the vesicles from degrading, it is advised to freeze the samples at 80 °C. Additionally, it is advised against repeatedly freezing and thawing the aliquots before to analysis
	Investigating EV composition	Sample replications as well as adjustment of the findings for multiple comparisons is essential

## EXTRACELLULAR VESICLE AND ENDOMETRIOSIS

Endometrial tissue-specific proteins and nucleic acids have been found to be present within EVs. Researchers have undertaken the investigation of potential biomarkers specifically linked to endometriosis within EVs. These biomarkers have the potential to be identified in bodily fluids such as blood, offering a noninvasive means of diagnosing endometriosis.

The peroxidation of lipoproteins by reactive oxygen species causes DNA damage in endometrial cells. In one study, researchers exposed both endometrial cancer cell lines and primary endometrial stromal cells to oxidized low-density lipoproteins (LDL) and found that this exposure caused DNA damage and increased levels of oxidative stress markers^[95]^. These oxygen species could be derived from the peritoneal fluid’s water and electrolytes such as sodium, potassium, and chloride, as well as glucose, proteins, and immune cells. Iron levels increase in the peritoneal cavity as hemoglobin breakdown occurs, leading to redox reactions as well. In an *in-vivo* study, it was stated that local inflammation causes lymphocyte recruitment and macrophage activation, which increases the secretion of cytokines that cause enzyme oxidation and promote endometrial cell growth^[96]^.

Recently, the immunological mechanism of endometriosis has drawn more interest, particularly because of the abundance of Extracellular vesicle (EV)--related endometriosis research. One study found that EVs may promote endometriosis by stimulating angiogenesis, proliferation, and inflammation in endometriotic lesions by analyzing the content of EVs isolated from patients with endometriosis^[97]^. Sun *et al.* suggest that peritoneal macrophages (MPs) may be involved in initiating the inflammatory response that results in endometriosis^[98]^. EVs produced from endometriosis ectopic stromal cells have the potential to make mice gain weight overall and reduce macrophage capacity for phagocytosis^[99]^. Some evidence suggests that macrophages collect in the peritoneal cavity of people with endometriosis, but whether they can clear displaced endometrial fragments is still unclear^[100]^. EVs produced from human endometrial stromal cells were observed to promote the formation of endometriotic lesions in mice^[101]^. Research suggests that elevated levels of circulating total cell-derived EVs in patients with deep-infiltrating endometriosis may cause immune system dysfunction, thereby promoting endometriosis development. Therefore, it has been demonstrated that EVs may help to promote endometriosis.

Excessive endometriotic tissue deposition on the extracellular matrix is the primary pathological feature of endometriosis, known as ectopic tissue fibrosis^[102]^. Laparoscopic diagnosis with a tissue sample is currently the gold standard for endometriosis^[103]^. Both methods of detection, however, are intrusive and potentially carry risks in the diagnosis of endometriosis. EVs obtained from cervicovaginal lavage and vaginal swabs have been shown in pre-clinical studies to be a novel, minimally invasive method for diagnosing several disorders of the reproductive tract, including endometrial disease^[104]^. It is speculation that needs additional research to confirm if it is true.

## ENDOMETRIAL CELL-DERIVED EXTRACELLULAR VESICLES AND THEIR SIGNIFICANCE

EVs from uterine biofluids play a critical role in various aspects of reproductive physiology. Conceptuses, uterine epithelia, and trophectoderm cells readily uptake EVs from uterine flushes, and progesterone stimulation promotes their release, carrying miRNAs that target the PI3K/AKT and BMP signaling pathways^[105]^. Pre-implantation EVs modulate apoptosis-related gene expression, while post-implantation EVs enhance adhesion molecule genes, exerting control over implantation^[106]^. In cows, exosomes and miRNAs are key players in affecting IVF embryo development and impairing embryo quality. Proteins in avian uterine fluid-derived EVs are vital for sustaining sperm activities^[107]^. Additionally, endometrial-derived EVs transport miRNAs that influence the embryo's transcriptome and genes involved in embryonic adhesion. These EVs contain various short RNAs crucial for endometrial remodeling and immune system regulation^[108]^. Furthermore, the transcriptomic cargo of uterine flush EVs reflects the changes during ovulation phases and endometrial tissue composition. These vesicles also hold predictive value for endometrial receptivity, embryo implantation, and implantation success in both fertile and infertile women^[109]^. In cell culture models, EVs from endometrial cells enhance trophectodermal adhesion and penetration into the endometrial epithelium, facilitating modifications in trophectoderm function^[110]^. Specific miRNAs within these EVs facilitate endometrial-embryo crosstalk and support embryo implantation. However, in cases of recurrent implantation failure, these EVs hinder trophoblast proliferation, migration, and invasion^[109]^. The secretion and miRNA screening of endometrial EVs are affected by the physiological conditions of the uterus^[111]^. Notably, EVs from patients with recurrent implantation failure impede blastocyst production, leading to reduced cell numbers in the embryo and compromised invasive capacity [Table 3]. Examining uterine aspirates allowed researchers to find the first indication of the existence of endometrial EVs^[111]^. Evidence supporting this hypothesis comes from the secretory phase, which showed samples collected from fertile women, where EVs were positive for tetraspanins CD9 and CD63^[105]^. Evidence that uterine EVs were secreted from the endometrial epithelial cells was provided by cell-surface marker immunostaining, which showed that EVs were localized on the apical surface of the luminal epithelial cells^[105]^. To understand the function of endometrial EVs in the implantation process, more experimental and scientific data are required.

**Table 3:** Recent significant discoveries on extracellular vesicles produced from endometrium

**SN**	**Major findings**
	**Source: EVs from Biofluid (uterine affluent)^[110]^**
	Conceptuses, uterine epithelia, and trophectoderm cells uptake uterine flush EVs
	Progesterone promotes the release of EVs from endometrial cells and the release of miRNAs found in EVs that target the PI3K/AKT and BMP signaling pathways
	Pre-implantation EVs up-regulate the expression of apoptosis-related genes, whereas post-implantation EVs up-regulate the expression of adhesion molecule genes. Uterine fluid EVs control implantation
	Exosomes and miRNAs are the primary mechanisms by which EVs from cows' uterus affect the development of IVF embryos and impairing molecule for IVF embryo quality
	Proteins found in EVs recovered from avian uterine fluid may be crucial for maintaining sperm activities inside the female genital tract
	MiRNAs from endometrial-derived EVs are transported by the endometrium and digested by the trophectoderm, affecting the transcriptome analysis of the embryo for genes implicated in embryonic adhesion
	Various short RNAs that are important in controlling endometrial remodeling pathways and immune system activities can be found in uterine flush EVs
	Uterine flush EVs' transcriptomic cargo represents the changes between the non-responsive and receptive phases of the ovulation as well as the endometrial tissue's RNA composition
	The endometrial receptivity, embryo implantation, and implantation success of uterine lavage EVs from fertile versus infertile women are all predicted
	**Source: EVs from endometrial cells models (cell culture)^[111]^**
	Human trophectodermal adhesion and penetration to endometrial epithelium were enhanced by endometrial-derived EVs. It was also demonstrated that transfer of endometrial EV cargo proteins to trophectoderm mediates modifications in trophectoderm function
	Specific miRNA found in endometrial-derived EVs may play a role in endometrial-embryo crosstalk and embryo implantation
	By limiting trophoblasts' ability to proliferate, migrate, and invade, endometrial EVs from patients with recurrent implantation failure interfered with their ability to perform
	The endometrium secretes EVs, and the uterus' physiological conditions affected by these EVs miRNA
	Recurrent implantation failure EVs prevent the production of blastocysts, which lowers the overall number of cells in the embryo and its capacity for invasion

In an *in vitro* study, it was shown that endometrial-derived EVs are regulated by hormones and menstrual cycles, and they have been shown to play a crucial role in endometrium blastocyst interactions, which are necessary for establishing pregnancy within the uterine cavity^[112]^. This investigation examined the proteomic profile of EVs modified by estrogen plus progesterone (to mimic the receptive endometrium) and estrogen (to mimic the proliferative phase) alone to determine the potential for endometrial-derived EVs to modify trophoblast function^[113]^. It was discovered that 254 proteins were concentrated in estrogen-influenced EVs, while 126 proteins were enriched in estrogen plus progesterone-influenced EVs^[114]^. It is interesting to note that the combination of estrogen and progesterone affected EV proteins that were connected to implantation modifications such as adhesion, migration, and invasion. Human trophoblast cells have been shown to take up EVs, causing them to change their characteristics and enhance their adherence when implanted after receiving the EVs. Focal adhesion kinase (FAK) signaling has been shown to play a role in this response^[114]^. Notably, FAK is a discovered mechanism in inner cell mass-trophectodermal cell communication, as well as between EVs and blastocysts^[115]^. Therefore, the results of this study support the hypothesis that endometrial exosomes play a significant role in mediating endometrial-embryo interactions in a uterine microenvironment model. Similarly, many studies have demonstrated the significance of endometrial EVs and the changes in their protein cargo that occur during the implantation process. Bovine intrauterine exosomal effects on conceptus implantation were investigated in one study^[116]^. This study demonstrated that pre-implantation EVs recovered from bovine endometrial epithelium differentially expressed many proteins compared to post-implantation EVs. Additionally, EVs collected from pre- and post-implantation periods of bovine uterine flushing fluids were subjected to protein analysis. The results of the investigation found that compared to EVs collected after implantation, EVs from the uterine lining prior to fertilization contained a significantly higher concentration of proteins such as caspases, Bcl-2 family protein, Death receptor, and ligands, p53, TNF-alpha associated with cell death^[116]^. Evidence like this may suggest that endometrial EVs have a paracrine effect on receptivity and implantation, though these alterations may also be the result of physiological changes in the epithelium. It appears that endometrial EVs have an important role in a variety of physiological and pathological processes related to implantation and fertilization. By providing a noninvasive biomarker for diagnosis and a novel therapeutic tool for promoting tissue repair and regeneration, EVs may offer a solution to the problem of infertility. EVs have been the subject of promising research as a clinical biomarker for applications such as liquid biopsies in diagnostics and therapy^[117,118]^, which may provide a solution to the problem of infertility.

## FUTURE SCOPE AND CLINICAL RESEARCH

To date, most studies have been conducted using *in vitro* or *in vivo* methods. Our knowledge of EVs and the reproductive cargo they transport was greatly advanced by studies in animal models. The complex and dynamic environment of the human reproductive system, however, cannot be currently replicated in *in vitro* research. There is a need for further epidemiologic research, particularly prospective studies that are guided by existing data to understand the connections between external exposures, EV cargo, and human-relevant reproductive outcome health. The mechanistic routes, including the specific EV cargo components thought to cause biological responses, are not as well understood, despite the abundance of evidence demonstrating that EVs and their cargos facilitate intercellular communication and have a major impact on female reproductive health. Few studies have been conducted to evaluate the proteomic, metabolomic, and RNA payloads using sensitive, unbiased techniques. In several studies that have found differentially expressed EV-miRNAs, possible mRNA targets are found using existing databases, and biological implications are inferred using ontological methods. Because of the gaps in our understanding of miRNAs and their targets, such preliminary findings are helpful but should be interpreted with caution. Ontological and route analyses are obstructed by problems such as incomplete or inaccurate annotations, a failure to take cell types into account, a subjectivity towards focusing on canonical pathways^[119,120]^, and the possibility of many ambiguous pathways that require extensive curation. Therefore, mechanistic research is required to assess the precise biological responses of the target tissue after implementing EVs with specific load profiles. Endometriosis is also linked with bacterial infection. There could be a potential association between a specific group of bacteria and endometriosis, a painful condition that impacts as many as 10% of women and girls in their reproductive years^[121]^. The bacterial genus *Fusobacterium* was found in the uterus of around 64% of those with endometriosis, and 7% without symptoms. This study was carried out in Japanese women (*n* = 155 subjects)^[122]^. This evidence supports the idea that bacteria-derived-outer membrane vesicles (OMVs) could be a potential area of interest for endometriosis treatment manipulation and vaccine development. The most recent study unravels how bacteria-derived-OMVs can be engineered and exploited for therapy and vaccine development^[123,124]^.

## CONCLUSION

Several studies on biosynthesis, composition, interactions with recipient cells, isolation techniques, and biological properties of EVs have been carried out since the discovery of their existence^[125]^. Therefore, it is critical to understand how EVs play a role in both physiological and pathological processes. For a successful pregnancy, it is important to maintain continuous molecular communication and this communication should rely on endocrine, paracrine, and autocrine components^[117]^. EVs could also be used as treatments and biomarkers. Studies are being done on how to prevent EVs from forming, releasing, and being absorbed by recipient cells, and how they might be used therapeutically via the introduction of modified or engineered EVs into biological systems^[126]^. Although many compounds produced from EVs have been identified as potential biomarkers, only a limited of them are suitable for therapeutic use. However, EVs made from synthetic or semi-synthetic materials have not yet been produced in the context of pregnancy. Although EVs and the components like miRNAs, mRNAs, and proteins in various reproductive biofluids have been shown to communicate with one another via their secreted EVs, further research is needed. It is critical to expand our knowledge of the biology of EVs in reproductive tissue to develop new ideas for enhancing fertility and understanding that EVs are a biological constituent with physiological and diagnostic implications in their establishment; hence, more research in this field is required.

## DECLARATIONS

### Copyright

© The Author(s) 2023.
